# Mobile app use by medical students and residents in the clinical setting: an exploratory study

**DOI:** 10.29173/jchla29562

**Published:** 2022-04-01

**Authors:** Karine Fournier

**Affiliations:** Research Librarian in Health Sciences and Medicine, Health Sciences Library, University of Ottawa, Ottawa, ON

## Abstract

**Introduction:**

Mobile devices and mobile applications facilitate access to clinical evidence at the point-of-care. Medical libraries play an important role in medical trainees' education, by subscribing to quality resources and by providing help and guidance on what apps to use. This study’s goal was to explore medical trainees' mobile applications use in the clinical setting to help inform collection development’s decisions and to provide insight on educational outreach. Perceived barriers and benefits of medical app use by clinical trainees was also explored.

**Methods:**

A brief online survey (English and French) was sent to all University of Ottawa clerkship medical students and residents. The questionnaire consisted of multiple choices, Likert-scale, and open-ended questions.

**Results:**

208 English and 9 French responses were received. UpToDate was the most frequently used app, followed by MedCalc, Spectrum (CHEO) and Medscape. Respondents used medical apps mostly before and after meeting with patients and rarely while interacting with patients. Main benefits identified of medical app use were helping with decision-making, quick access to trustworthy clinical information, help with diagnosis and treatment options (e.g. medication dosage, drug interaction). Main barriers identified were costs, appearing unprofessional, lack of Canadian content and spotty hospital WiFi.

**Conclusion:**

Libraries' involvement in providing access to trustworthy clinical resources to medical trainees is important to help shape trainees' development as medical professionals. Outreach to learners in the clinical setting is crucial to educate on what apps are available to them through the library collection.

## Introduction

Mobile devices and mobile applications (apps) allow medical students and residents to access clinical evidence at the point-of-care. In a recent study, all medical students completing their first year of clinical placement surveyed owned a smartphone and almost all students were using technology daily for educational purposes [1]. The term “m-learning” partly captures this phenomenon and is defined as “learning via a mobile device such as a tablet or smartphone” [2]. Crompton acknowledges the year 2005 as when m-learning, or mobile learning, became a recognized term. She also identified the four pillars of m-learning as “pedagogy, technological devices, context, and social interaction” [3].

Apps that are considered “medical” are found in the thousands [4]. Not all apps are created equal, and some are perceived more trustworthy than others by medical students for clinical decision support [5]. Bradley-Ridout et al. performed a crossover research study comparing UpToDate and DynaMed on accuracy, time to answer, user confidence, and user satisfaction [6]. Participants were more confident and satisfied in using UpToDate, although accuracy was the same between the two apps and time to answer was higher with DynaMed. Participants were all, except for one, previous users of UpToDate compared to DynaMed, which could explain participants’ higher comfort and confidence levels using UpToDate [6].

Clarke et al. observed that most medical students used their mobile devices for entertainment, communication, and social media in addition to m-learning, while completing their clinical placement [1]. This non-work-related mobile device use led patients and other staff to view mobile device use to be unprofessional. Researchers have observed that many medical students believed that using their mobile devices in front of patients gives the impression of being less engaged [5,7]. However, they found that when students disclosed their reasons for using their devices to hospital staff or patients, they prevented negative reactions. An additional barrier to the use of apps at the point-of-care are outdated hospital rules regarding mobile device use: “many hospitals continue to regulate the use of mobile phones through formal policies, restricting their use within certain parts of the hospital or within departments” [7]. A recent systematic review looking at mobile device use by medical trainees calls for clearer, more explicit institutional policies regarding the use of mobile devices in clinical setting [8].

Despite these barriers, many studies have highlighted the positive and important impact of mobile device usage by medical trainees. These include enhanced learning by providing access to important clinical knowledge at the point-of-care [1,5,9-10], and clinical decision-making support by offering crucial timely information when interacting with patients [7,11-12].

Medical libraries subscribe to many online resources, which often also include an app version. A 2014 pan-Canadian study found that participants (a mix of students, residents and faculty members) mostly used mobile devices to find drug information, to make clinical calculations, and to take notes [13]. When asked their favourite resources, participants listed: UpToDate, Epocrates, Medscape, eMedicine, Lexicomp, PubMed, DynaMed, PEPID, a medical calculator, Micromedex and the Internet [13]. They also asked participants what barriers they faced accessing medical information on mobile devices. The main barriers identified were wireless access in the hospital or clinic and knowing what resources are available to them.

A 2019 [14] study surveyed clerkship students asking which point-of-care resources they would use to answer a patient question. They asked participants to select one or more of the four resources listed (DynaMed, Epocrates, UpToDate, and VisualDX). The author reported that most students selected a combination of the following point-of-care tools: UpToDate, DynaMed and Epocrates, and a portion of respondents selected UpToDate solely.

At the University of Ottawa, there has been a constant increase in price in the last few years for subscriptions, and although there is a healthy collection budget, choices have to be made every year on what resources should be kept or cut. Knowing what apps are used by residents and medical students can help inform library collection decision-making and what educational outreach is needed. It appears no studies have explored mobile app use by medical trainees in Canada in the last eight years.

Therefore, the purpose of this article is to explore the practices of clerkship students (medical students in years 3 and 4) and residents using mobile medical apps in clinical settings. Of particular interest was exploring the perceived usefulness, barriers, and benefits of using these apps.

Research questions included:

1. Do residents and clerkship medical students use clinical mobile apps?
2. If so, which ones do they use, and how do they rate them for helping to answer clinical questions?
3. What are the perceived benefits and barriers of using mobile apps in the clinical setting?

It is hoped that the results from this study will inform what and how mobile apps are used by medical trainees on a local level, and in turn, influence library collection decisions to better reflect trainee’s needs and support educational outreach programming.

## Methods

This study was approved by the University of Ottawa in December 2019 (Ethics File Number: H-12-19-5245). The brief bilingual (French and English) survey was comprised of 14 questions, including multiple choice, Likert-scale, and open-ended questions, (Online Supplement, Appendix 1). Each question was optional. SurveyMonkey was used to disseminate the survey. Development of the survey was informed by the literature cited in this paper and feedback received by colleagues and the University of Ottawa Institutional Research and Planning office with their extensive knowledge on how to best survey student populations. If participants wished to participate in a draw to win a $100 Amazon gift card, they could provide their name and email. Once the draw was completed, identifying information was destroyed.

The questionnaire was emailed to potential respondents and was open from January 13^th^ to February 29^th^, 2020. One email reminder was also sent. Once the survey was closed, answers were exported from SurveyMonkey to Microsoft Excel, which was used for descriptive statistics. The author cleaned the data, and merged the French and English answers.

Answers to open-ended questions were brief which made coding and identifying emerging themes possible by hand. No specialized software was required.

It is important to note that when respondents were asked what top three mobile apps they use for decision-making in the clinical setting this question was kept open-ended as not to lead respondents’ answers with preselected choices. Their answers were then compiled to discern the number of times each app was listed by participants, regardless of their position in the top three.

## Results

208 English and 9 French responses were received. All responses were included in the final analysis, including unfinished questionnaires. Respondents’ demographic distribution was as follows: 39 medical students, and 149 residents answered our survey. 29 participants did not answer the demographic questions, but their answers were included in the analysis to help enrich the results and inform the research questions. As incomplete responses were permitted participants were under no-obligation to answer the demographic questions. Taking into consideration the participants who self-identified as medical students or residents, the response rate was 11% (n=39) for medical students (years 3 and 4), and 21% (n=149) for residents.

Participants were asked if they use their mobile device in the clinical setting. 88% (n=191) of the survey’s participants answered, “all the time” or “often”, 11% (n=23) stated “sometimes” and 1% (n=3) “never”. The participants who answered “never” were thanked for their participation and the questionnaire ended for them at that point.

**Fig. 1:**
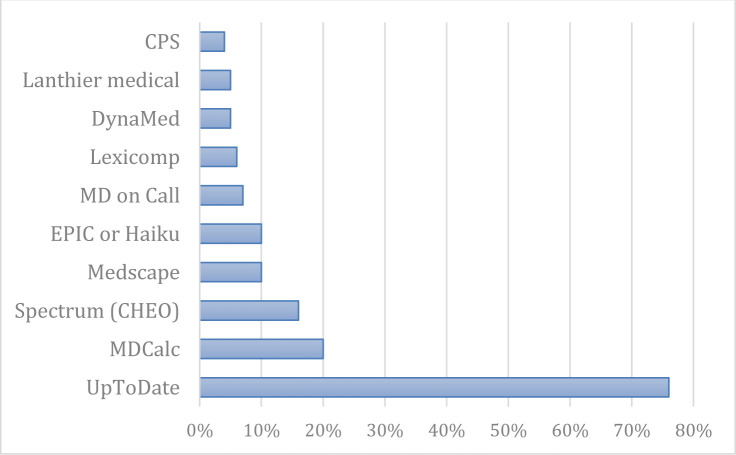
Preferred medical apps for clinical decision support. (N=214, 99% of respondents answered this question.)

As previously mentioned, the number of times the following apps were mentioned by participants was calculated, regardless of their position in the top three. UpToDate was mentioned by 76% (n=166) of participants, followed by MDCalc (20%, n=43), Spectrum (16%, n=36), Medscape (10%, n=22) and MD on Call (7%, n=15). Lexicomp and DynaMed were respectfully mentioned by 6% (n=14) and 5% (n=13) of participants (see figure 1). (Spectrum is an infectious diseases app created by the Children's Hospital of Eastern Ontario (CHEO) Antimicrobial Stewardship Committee. For more information: https://app.spectrum.md/.) Many other apps were mentioned only by a handful of participants, such as Epocrates (1%, n=3), and didn’t make the cut to the top 10. A complete list of apps mentioned can be found in Online Supplement, Appendix 2.

When asked what their reasons were for choosing these specific mobile apps, respondents answered “being free” (48%, n=105) and “suggested by a friend or colleague” (50%, n=109) the most.

**Fig. 2:**
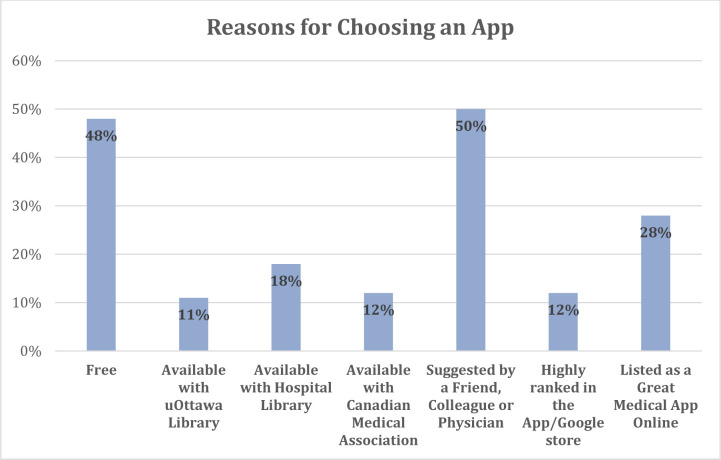
Reasons for choosing an app (N=192, 88% of respondents answered this question.)

Another open-ended question inquired into what apps they use for drug information. The three main apps mentioned were UpToDate (42%, n=91), Lexicomp (14%, n=31) and CPS (8%, n=18).

**Fig. 3:**
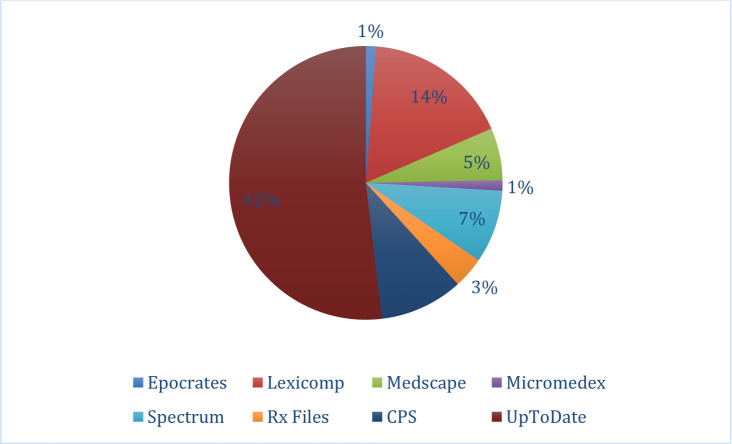
Apps used for drug information. (N=188, 87% of respondents answered this question.)

We asked participants the frequency of which they used specific mobile apps. UpToDate was used “often” or “all the time” by 74% (n=157) of respondents. There was a drop in usage for all other apps. Lexicomp and CPS were used “often” or “all the time” by 28% (n=62) and 8% (n=17) of our respondents.

**Table 1:** Frequency of app use. (N=193, 89% of respondents answered this question.)

Top 5	App	Never	Sometimes	Often	All the time
**1**	**UpToDate**	8%	7%	16%	56%
**2**	**Lexicomp**	37%	21%	19%	9%
**3**	**CPS**	57%	22%	5%	3%
**4**	**DynaMed**	56%	26%	5%	0
**5**	**WebMD**	56%	20%	5%	2%

Participants were also asked to rate these apps in helping answer clinical questions with “1” being not helpful at all for clinical decision support, and “5” being extremely helpful for clinical decision support. Participants could choose “don’t know”, which many selected.

**Table 2:** Top 5 apps with average rating. (N=184, 85% of respondents answered this question.)

Top 5	App	Average Rating (1-5)
1	UpToDate	4.6
2	LexiComp	4
3	DynaMed	3.1
4	CPS	3
5	WebMD	2,6

Participants were asked what apps the library should subscribe to, to help with their learning. Respondents mainly answered UpToDate (70%, n=112), followed by Lexicomp (9%, n=14), CPS (3%, n=5) and Dynamed (2%, n=4).

Participants were also asked when they use medical apps: before, during or after their interactions with patients. Most participants used medical apps before (77%, n=168) and after (82%, n=178) meeting with patients, and only a fraction while interacting with patients (27%, n=27).

**Fig. 4:**
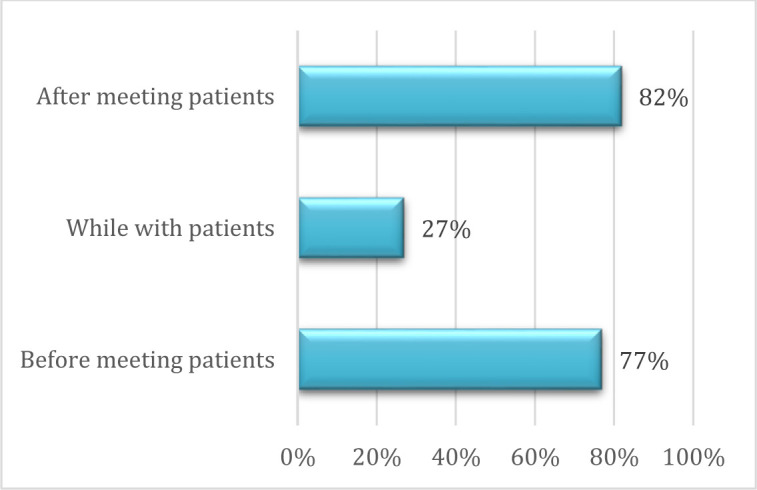
Timing of App Use. (N=187, 86% of respondents answered this question.)

Participants had the opportunity to share what they believed to be the benefits and barriers of using medical apps in clinical settings in an open-ended question. 85% of participants (n=185) answered.

Four themes emerged regarding benefits:

- Quick and easy access to evidenced-based, reliable and updated clinical knowledge;
- Checking medication doses and interactions;
- To prepare before meeting with patients, form a clinical plan, or help differential diagnosis;
- Better patient care, safer prescribing, reducing medical errors.

Noteworthy quotes that represent well respondents’ answers include: “Mobile applications let me access resources more easily and readily than poor hospital infrastructure (not enough computer terminals, outdated or slow and frequently crashing)”; “It's impossible to be knowledgeable about all areas of medicine and apps help tremendously with problems outside my area of expertise”; “Learn as you go, make more accurate plans before discussing with supervisors, understand your patients better”; “Helps me feel more prepared”.

Five themes emerged in relation to participant-identified barriers:

- Negative perception from patients and supervisors (e.g. appearing rude or unprofessional);
- Costs;
- Spotty hospital WiFi;
- Lack of Canadian content;
- Data plan limitations, phone’s storage limit, battery life.

Respondents stated the following: “Optics of being on phone appearing like you don't know things you maybe should”; “Stigma of pulling out your phone in clinic”; “I avoid using my phone as I worry that staff and residents will assume I am not using it for clinical purposes”; “Sometimes it takes a while to pull up, poor internet connection; patients may not feel confident in your medical knowledge if used in front of them”; “cost of some of the useful apps.”

## Discussion

The first research question addressed whether residents and clerkship medical students use clinical mobile apps. 99% (n=214) of participants identified that, yes, they used medical apps for clinical decision support. This led to the question of what apps are being used by these Ottawa based clerkship students and residents and how they rate them. UpToDate as the preferred app was surprising since only a few of the hospital libraries in the region subscribe to it, and it is not available through the University of Ottawa collection. Previous research is conflicting. Some have demonstrated the popularity of UpToDate among students, residents and physicians [13, 14]. Whereas the pan-Canadian study [13], Hoogland [14] and Nuss [12] identified Epocrates as one of the main apps used by participants. However, only 1% (n=3) of University of Ottawa clerkship students and residents identified Epocrates as a preferred app.

At the University of Ottawa, the collection decision was made to subscribe to DynaMed to provide equivalent point-of-care clinical information to clerkship learners and residents as UpToDate, but very few participants demonstrated using DynaMed. The University of Ottawa also subscribes to CPS but not to Lexicomp, and the results showed that more people were using Lexicomp compared to CPS. These results were surprising in a large part because the library provides training to third year medical students and first year family medicine residents on different apps available to them via the library.

Furthermore, since the results showed that clinicians’ opinions appear to influence learner’s app selection, one could tentatively argue that UpToDate and Lexicomp have been the resource of choice by many clinicians since their debut - even if competitors have risen in the last few years (e.g. DynaMed). As one participant commented “I wish we got a legitimate tutorial on how to use the apps during clerkship - the ones I use are the ones doctors showed me they like” which sums this up well. The library subscribes to resources, which learners don’t use either because they didn’t know they had access to them, or because they use the ones their supervisors have suggested.

Participants also rated UpToDate and Lexicomp higher than DynaMed and CPS overall. This is presumably because since both UpToDate and Lexicomp were used a lot more by participants than DynaMed and CPS, they had little experience of these resources’ functionalities. This presumption aligns partly with Bradley-Ridout et al. findings, who studied UpToDate and DynaMed use and comfort level by residents [6]. Anecdotally, one participant also noted that CPS was difficult to navigate. There were also a few participants that suggested the library should subscribe to DynaMed and CPS. This demonstrated their lack of knowledge of the apps available through the library to them identifying a need for enhanced outreach.

The last research question was intended to find out the perceived benefits of and barriers to the use of apps in the clinical setting. As shown in the results, respondents are preoccupied by the negative perception of unprofessional behaviour when using their mobile device in the clinical setting. This aligns with recent findings by Clarke and Dimond [1,7]. However, these results differ from what was identified as the main barrier listed by residents and clerkship students in the pan-Canadian study from 2014, which were WiFi issues and lack of knowledge on what resource to use [13]. However, WiFi issues were also one of the main barriers identified by University of Ottawa respondents.

Learners’ concerns regarding looking unprofessional are perhaps the consequence of a generational gap. It is clear however that this is a barrier for these learners to improving their clinical knowledge and perfecting their diagnosis skills while in the clinical setting where mobile devices are a quick method of accessing reliable clinical knowledge. Educating hospital staff and patients might be the key to solving this issue by raising awareness of mobile device use for educational purposes (m-learning) and not for pleasure or entertainment. A recent study found that: “…newly qualified doctors recognized that the way they were using their phone was important. By sharing the resources with patients, their mobile phone facilitates patient centered care, but it also avoided the potential for criticism and uncertainty. Providing explanation, to patients or ward staff, as to why a mobile phone was being used within a work space usually enabled the doctors to proceed with confidence” [7].

Another obstacle highlighted by participants is the cost of medical apps. In the Ottawa region and in Canada, hospital library closures have and continue to occur, and those that remain often have limited budget preventing these libraries from providing institutional access to point-of-care apps [15]. As for the University of Ottawa library, the budget enables subscriptions to point-of-care resources and apps however not to all of them. Therefore, some app costs are transferred to the learner, who will have a median debt of $100,000 by the time they finish medical school [16]. Some participants also stated the lack of Canadian content as a barrier, but as very few participants used Canadian apps readily available to them, it begs the question if the problem is a lack of knowledge of app availability and therefore educational outreach.

As for the benefits identified by our respondents, (e.g., access to reliable clinical information, dosage checking, help forming a clinical plan and help with differential diagnosis); they align with what has been reported by previous studies [1,5,7,9-12]. No new benefits have been identified in this study.

Although this research project experienced limitations , it is hoped that the results of this study will help inform current research in the Canadian context as well as decision making in various library areas such as collection development, education and outreach, promotion etc.

## Conclusion

These results showed that even if there are some library educational sessions on point-of-care apps already in place for medical students and residents, learners are not using the resources available through the library collection. As stated earlier, the University of Ottawa Library has a healthy budget, but still cannot subscribe to all point-of-care apps. As a next step, the library will be looking at re-evaluating and exploring apps that would best suit learners. As well as re-imagining educational sessions and putting the emphasis on Canadian-content, and apps available to them. Providing outreach to additional resident groups, and offering educational training to hospital staff and physicians on the value of m-learning and on Canadian-centric apps could perhaps help re-orient learners’ app selection and use in return.

Furthermore, the results showed that participants’ use medical apps very frequently before and after meeting with patients and sometimes during patient consults. They also see the benefit of using these apps for rapid access to trustworthy clinical information. It seems fair to say that mobile devices and point-of care mobile apps have their place in the learner’s toolkit. Although there are barriers to their use, point-of-care apps are useful to learners in the fast-paced clinical environment. Education is key and remains important in shaping learners’ habits in what resources to use. Future research could focus on hospital staff and clinician’s perceptions of mobile device use by medical trainees to better understand the challenges learners encounter and learn how to help change these negative perceptions. Also, exploring why learners choose one app over another would perhaps provide a better understanding of apps’ selection by medical trainees.

### Limitations

This study provides a current picture of medical app use by learners in the clinical environment, in Ottawa, but the findings do have limitations. A relatively low response rate and having surveyed members of only one institution render the extrapolation of results to all medical learners in Canada difficult. Demographic details were asked at the end of the survey, and since unfinished questionnaires were included in the analysis this resulted in 29% of participants not disclosing if they were a medical student or a resident. This prevented further division of the results by demographic.

## Supplementary Material


