# Supplementary material for: Mobile app use by medical students and residents in the clinical setting: an exploratory study
Source: J Can Health Libr Assoc. 2022 Apr 1;43(1):3–11. doi: 10.29173/jchla29562 (PMC9359684; doi:10.29173/jchla29562)
Supplement: Supplementary file 1 [file JCHLA-43-003-s001.pdf]

Appendix 1: Questionnaire

1. How frequently do you use your mobile device (smartphone, tablet) in the clinical setting? *Multiple choices provided: 1. Never, 2. Sometime, 3. Often, 4. All the time*. Respondents answering “Never” will exit the survey.

2. What are your top three preferred medical applications (apps) for clinical decision support?

3. What made you decide to use these three apps specifically? (You can choose more than one answer):
    a. It was free
    b. It was available through the University of [XX] Library
    c. It was available through the local hospital library
    d. It was available through the Canadian Medical Association
    e. It was suggested by a friend, a colleague, or a physician
    f. It was highly ranked in the app store/google store
    g. It was listed as a great medical app online
    h. Other:

4. Which app(s) do you use for drug information?

5. Are you using the following apps... *Multiple-choices provided: 1. Never, 2. Sometime, 3. Often, 4. All the time*

    a. 5-Minute Clinical Consult
    b. AccessMedicine
    c. Diagnosaurus
    d. DynaMed
    e. Epocrates
    f. Isabel
    g. Lexicomp
    h. Micromedex
    i. PEPID
    j. CPS
    k. RxFiles
    l. UpToDate
    m. Visual Dx
    n. WebMD
    o. Other:

6. Please rate the following apps, where "1" means not helpful at all for clinical decision support, and "5" means extremely helpful for clinical decision support. A*pps which received*

the answer “Never” in question 5 won’t appear in this question. The option "do not apply" will also be available.

a. 5-Minute Clinical Consult
b. AccessMedicine
c. Diagnosaurus
d. DynaMed
e. Epocrates
f. Isabel
g. Lexicomp
h. Micromedex
i. PEPID
j. CPS
k. RxFiles
l. UpToDate
m. Visual Dx
n. WebMD
o. Other:

7. What medical apps should the University of [XX] Library subscribe to help you as a learner in the clinical environment?

8. Do you use medical apps… (you can select more than one answer)?
    a. Before meeting with patients
    b. While interacting with patients
    c. After meeting with patients
    d. None of the above

9. What are the ***benefits*** of using medical apps in clinical settings? You can list more than one benefit.

10. What are the ***barriers*** of using medical apps in clinical settings? You can list more than one barrier.

11. Are you…
    a. A medical student
    b. A resident
    c. Other:

12. *If the respondent answered "medical student":* What year?
    a. Year 1
    b. Year 2
    c. Year 3
    d. Year 4

13. *If the respondent answered "resident":* Which program?

Anatomical Pathology

Anesthesiology

Cardiac Surgery

Dermatology

Diagnostic Radiology

Emergency Medicine

Family Medicine

General Surgery

Hematological Pathology

Internal Medicine

Medical Genetics

Medical Microbiology

Adult Neurology

Neurosurgery

Nuclear Medicine

Obstetrics and Gynecology

Ophthalmology

Orthopedic Surgery

Otolaryngology-Head & Neck Surgery

Pediatrics

Pediatric Neurology

Physical Medicine & Rehabilitation

Plastic Surgery

Psychiatry

Public Health and Preventive Medicine

Radiation Oncology

Urology

Vascular Surgery

Other:

14. Do you have any additional comments?

Appendix 2: Complete List of Apps Used by Medical Trainees

ANSO

Antibiogram

Antiinfective Guidelines

AO Surgery Reference

ASRA Coags

Bc guidelines

Billing app

BNF

Bugs and Drugs

Calcium correction

Calculate by QxMD

Canada sti

Canadian Cardiovascular Society Guidelines

CDH guidelines

Community Acquired Infections

Compendulum of Pharm (Canada)

DSM 5

Dynamed

e-Anatomy

EPIC

Epocrates

Euroscore II

Evernote

Explorer

EyeWiki

FM notes

FP notebook

FPnotes

Google

Google scholar

Haiku

Hospitalist handbook

iCCS

Infectious disease guidelines

inkling

internet

Journal Club

Lanthier

Learn ent

Lexicomp

lymph nodes

MD on Call

MDCalc

Med RX

Medbullets

medical journal

Mediquations

Medscape

Merck Manual

MicroMedex

Microsoft OneNote

MUMS antibiotic guidelines

Nbn2

NCCN Guidelines

Neomate

Nerve whiz

Neuroscience Education Institute Prescribe

Nomanclature based naming

Notability

Nuance powermic

OMA app

On-Call

one note

OnExam

Orthobullets

Orthoguidlines

Pedi STAT

PowerMic mobile

Prescribe Smart

Primary care rap

Pubmed

Quick EM

QxMD

Radiology assistant

Radiopaedia

RCH ClinicalGuidelines

Read by QxMD

Resident on call

Rku compact

Rx files

CPS

Safari

Sanford antimicrobial guide

Spectrum

STI government of Canada app

Switch RX

Textbooks

The orange book

Thrombosis Canada

trekk

u central

UpToDate

Vumedi

Web browsing

WikiEM
